# Roles of mTOR Signaling in Tissue Regeneration

**DOI:** 10.3390/cells8091075

**Published:** 2019-09-12

**Authors:** Xiangyong Wei, Lingfei Luo, Jinzi Chen

**Affiliations:** Laboratory of Molecular Developmental Biology, School of Life Sciences, Southwest University, Beibei, Chongqing 400715, China; xiangyongwei2016@outlook.com

**Keywords:** mTOR signaling, metabolism, tissue regeneration, neuron, muscle, liver, intestine

## Abstract

The mammalian target of rapamycin (mTOR), is a serine/threonine protein kinase and belongs to the phosphatidylinositol 3-kinase (PI3K)-related kinase (PIKK) family. mTOR interacts with other subunits to form two distinct complexes, mTORC1 and mTORC2. mTORC1 coordinates cell growth and metabolism in response to environmental input, including growth factors, amino acid, energy and stress. mTORC2 mainly controls cell survival and migration through phosphorylating glucocorticoid-regulated kinase (SGK), protein kinase B (Akt), and protein kinase C (PKC) kinase families. The dysregulation of mTOR is involved in human diseases including cancer, cardiovascular diseases, neurodegenerative diseases, and epilepsy. Tissue damage caused by trauma, diseases or aging disrupt the tissue functions. Tissue regeneration after injuries is of significance for recovering the tissue homeostasis and functions. Mammals have very limited regenerative capacity in multiple tissues and organs, such as the heart and central nervous system (CNS). Thereby, understanding the mechanisms underlying tissue regeneration is crucial for tissue repair and regenerative medicine. mTOR is activated in multiple tissue injuries. In this review, we summarize the roles of mTOR signaling in tissue regeneration such as neurons, muscles, the liver and the intestine.

## 1. Introduction

In the 1970s, a new antifungal, was discovered in soil samples on the Polynesian island of Rapa Nui, which was isolated from *Streptomyces hygroscopicus* and called rapamycin [1,2]. Afterwards, FK506-binding protein 12 (FKBP12) was found to repress cell growth and proliferation [3]. During the 1990s, the target of rapamycin (TOR) and the mammalian target of rapamycin (mTOR) were discovered in yeast and mammals respectively [2]. Brown et al. reported that mTOR is a target of the FKBP12-rapamycin complex [4]. mTOR is a serine/threonine protein kinase, which recruits other proteins to form two different complexes, named mTOR complex 1 (mTORC1) and complex 2 (mTORC2). mTOR is conserved in the evolution from yeast to mammal [1]. mTORC1 and mTORC2 contain the same subunits: mTOR, mammalian lethal with Sec13 protein 8 (mLST8) and DEP domain-containing mTOR-interacting protein (DEPTOR). However, regulatory-associated protein of mTOR (Raptor) and 40 kDa proline-rich Akt substrate (PRAS40) are specific to mTORC1, while rapamycin-insensitive companion of mTOR (Rictor), Protor1/2 and mammalian stress-activated protein kinase(SAPK)-interacting protein 1 (mSin1) are specific to mTORC2 [1,5]. mTOR signaling plays crucial roles in the regulation of cell growth, metabolism, cell survival and migration. In response to growth factors, energy, amino acid, and oxygen, mTORC1 controls cell growth and metabolism through mRNA translation, synthesis of protein, lipid and nucleotide, and repression of catabolic processes such as autophagy [6]. The ribosomal S6 kinase (S6K) and eIF4E-binding protein 1 (4EBP1) are the main effectors of mTORC1. Unlike mTORC1, studies on mTORC2 are limited. mTORC2 mainly controls cell survival and migration through phosphorylation and activation of the downstream-effectors SGK1, Akt, and the PKC kinase families [5]. The mTORC2 is an effector of the insulin/PI3K pathway and is a key regulator of Akt [5,7]. mTOR signaling is the central pathway in response to the environment, and the disruption of mTOR signaling is associated with developmental defects, cancer, neurodegenerative diseases, type 2 diabetes, autoimmune diseases, and aging-related diseases [8,9,10,11,12]. Thus, mTOR is therefore a therapeutic target of these diseases [13].

Tissue damage caused by trauma, diseases, and aging, etc. can result in organ dysfunction. Afterward, tissue regeneration is critical for the restoration of organ functions and maintenance of homeostasis [14]. In adult humans, although the regenerative capacity of some organs, like the central nervous system (CNS) and heart, is weak, other organs, including the liver, intestines, muscles, and skin, do maintain the intrinsic ability to regenerate [15]. The key reasons why different organs obtain distinct regenerative capacities and different species obtain distinct regenerative capacities in the same organ remain to be elucidated. So, mechanistic insights into tissue regeneration are essential for tissue repair and regenerative medicine [14]. mTOR is one of the central regulatory signaling pathways between injuries and physiological reactions such as tissue regeneration. For example, in the CNS with very weak regenerative capacity, activated mTOR through the inactivation of PTEN (phosphatase and tensin homolog) or TSC1 (tuberous sclerosis complex 1) can robustly promote axonal regeneration [16]. mTOR is also vital in the regeneration of the intestines, liver and muscles.

In this review, we first briefly describe the structures, regulatory mechanisms, and physiological functions of mTORC1 and mTORC2. Then, we put our efforts toward summarizing the roles of mTOR signaling in the regeneration of neurons, muscles, the liver, and intestine. At the end, the development strategy of tissue-specific agonist or inhibitor of mTORC1 in regenerative medicine is discussed.

## 2. The Structure and Regulation of mTORC1

**Figure 1:**
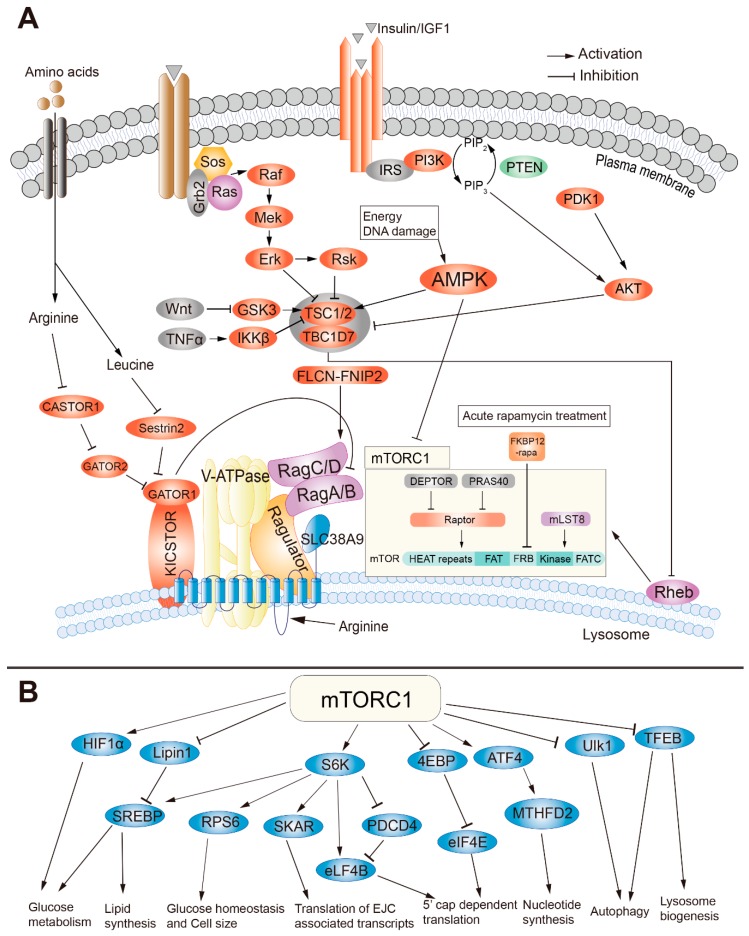
The regulatory mechanism and function of the mammalian target of rapamycin complex 1 (mTORC1). (**A**) The structures and regulatory mechanism of mTORC1. (**B**) The downstream functions of mTORC1.

mTOR is a serine/threonine protein kinase and a member of the PI3K-related kinase (PIKK) family, which forms the mTORC1 and mTORC2 complexes with other proteins [17,18]. The mTORC1, a heterotrimeric protein kinase, is mainly composed of three core components including mTOR, Raptor, and mLST8 [19,20,21,22]. mTORC1 also contains two inhibitory subunits, PRAS40 and DEPTOR [23,24,25]. After acute rapamycin treatment, the FKBP12-rapamycin complex binds to the FKBP12-rapamycin-binding (FRB) domain of mTOR and blocks mTORC1 activation [26] (Figure 1A). The mTORC1 plays important roles in metabolism and cell growth in response to nutrients and is regulated by many factors including growth factors, amino acids, energy, oxygen, and DNA damage [1,27]. Insulin/insulin-like growth factors (IGFs) inhibit the TSC complex, an inhibitory heterotrimeric complex of mTOR containing TSC1, TSC2, and Tre2-Bub2-Cdc16 (TBC) 1 domain family, member 7 (TBC1D7) [28], thus activating mTORC1. This mTORC1 activation is dependent on the Akt-mediated phosphorylation of TSC, which dissociates TSC from the lysosomal membrane [29]. The Ras signaling activates mTORC1 through extracellular signal-regulated kinase (Erk) and p90^RSK^, both of which phosphorylate and inhibit TSC2 [30] (Figure 1A). It is worth mentioning that Ras homolog enriched in brain (Rheb) is indispensable for mTORC1 activation. Some papers reported that Rheb activates mTORC1 through interruption of the FKBP38-mTOR interaction or directly binding to mTOR [31,32], however, the detailed mechanisms underlying activation of mTORC1 by Rheb remain to be fully elucidated.

The mTORC1 activation is closely related to the variation of amino acid concentrations. Different types of amino acid stimulate mTORC1 through different sensors. For example, cytosolic leucine, cytosolic arginine, and the lysosomal arginine are sensed by Sestrin2, CASTOR1 (Cellular Arginine Sensor for mTORC1) complex, and a candidate lysosomal amino acid sensor SLC38A9, respectively [1,33,34,35,36]. Amino acids activate mTORC1 through an amino acid sensing cascade involving the vacuolar H^+^-ATPase (v-ATPase), RAG GTPases (small guanosine triphosphatases) and Ragulator. Unlike other stimulators, mTORC1 activation by amino acids is independent of the TSC-Rheb signaling axis [37] (Figure 1A). In contrast to leucine and arginine, glutamine also promotes mTORC1 activation, which is dependent on the related Arf family GTPases rather than Rag GTPase [38]. Folliculin-FNIP2 (folliculin interacting protein 2) complex, a Rag-interacting protein with GAP (GTPase-activating protein) activity for RagC/D, was recently reported to activate mTORC1 in the existence of amino acids [36,39]. Except cytosolic arginine, leucine and lysosomal arginine, whether other amino acids activate mTORC1, and the identity of their sensors remains unknown. Furthermore, energy, oxygen, and DNA damage negatively regulate mTORC1 through AMPK (5’ AMP-activated kinase), which indirectly inhibits mTORC1 activation via phosphorylation of TSC2 or direct phosphorylation of Raptor [40,41,42]. Moreover, both wingless-type MMTV integration site family (Wnt) signaling and tumor necrosis factor α (TNFα) activate mTORC1 through inhibition of TSC1 [43,44].

The activated mTORC1 enhances protein synthesis through direct phosphorylation of the ribosomal S6 kinase (S6K) and 4E-BP1 [45]. Then, the phosphorylated S6K (pS6K) promotes mRNA translation initiation through phosphorylation and activation of eIF4B, a positive regulator of the 5′ cap-binding eIF4F complex, and promotion of the degradation of PDCD4 (programmed cell death protein 4), an inhibitor of elF4B [46,47]. pS6K also regulates glucose homeostasis and cell size through phosphorylation of ribosomal protein s6 (rps6) [48]. Moreover, the interaction of pS6K and SKAR (S6K1 Aly/REF-like substrate) improves the translation efficiency of spliced mRNAs [49]. The phosphorylated 4E-BP1 dissociates its binding to eIF4E, which allows eIF4E to join in the eIF4F complex together with eIF4G, thus permitting the cap-dependent translation [50]. All the regulations above finally promote protein synthesis. The mTORC1-dependent anabolism is mediated by phosphorylation of S6K, inhibition of lipin1, an inhibitor of lipid synthesis, [51] and activation of ATF4 (activating transcription factor 4), a promoter of nucleotide synthesis [52]. mTORC1 also augments the glycolytic pathway through increasing the translation of hypoxia inducible factor 1α (HIFα), which drives the expression of phospho-fructo kinase (PFK) [53]. Furthermore, mTORC1 suppresses the catabolism such as autophagy and lysosome biogenesis through phosphorylation of ULK1 (unc-51 like autophagy activating kinase 1) and the transcription factor EB (TFEB) [54,55] (Figure 1B). In conclusion, mTORC1 regulates cell growth and metabolism in response to environmental inputs such as growth factors, nutrients, and DNA damage. It plays significant roles in development, physiological processes, and diseases.

## 3. The Structure and Regulation of mTORC2

Like mTORC1, mTORC2 also contains mTOR and mLST8 subunits. But Raptor in mTORC1 is replaced by Rictor in mTORC2 [56]. mTORC2 also includes DEPTOR, the regulatory subunits mSin1 and Protor1/2 [25,57,58]. mTORC2 can be impeded by prolonged rapamycin treatment [59]. Unlike mTORC1, the upstream and downstream activity of mTORC2 are not well-defined. mTORC2, as an effector of insulin/PI3K signaling, is inhibited by the pleckstrin homology domain of mSin1 when there is a lack of insulin. This autoinhibition by mSin1 is relieved upon its binding to phosphatidylinositol (3,4,5)-trisphosphate (PIP3) on the plasma membrane [7]. Akt activates mTORC2 through phosphorylation of mSin1 at T86, in turn the activated mTORC2 stimulates Akt through phosphorylation of Akt at S473, which forms a positive feedback regulatory loop [5,60]. In contrast to Akt, S6K suppresses mTORC2 via promoting the degradation of insulin receptor substrate 1 (IRS1) [61].

**Figure 2:**
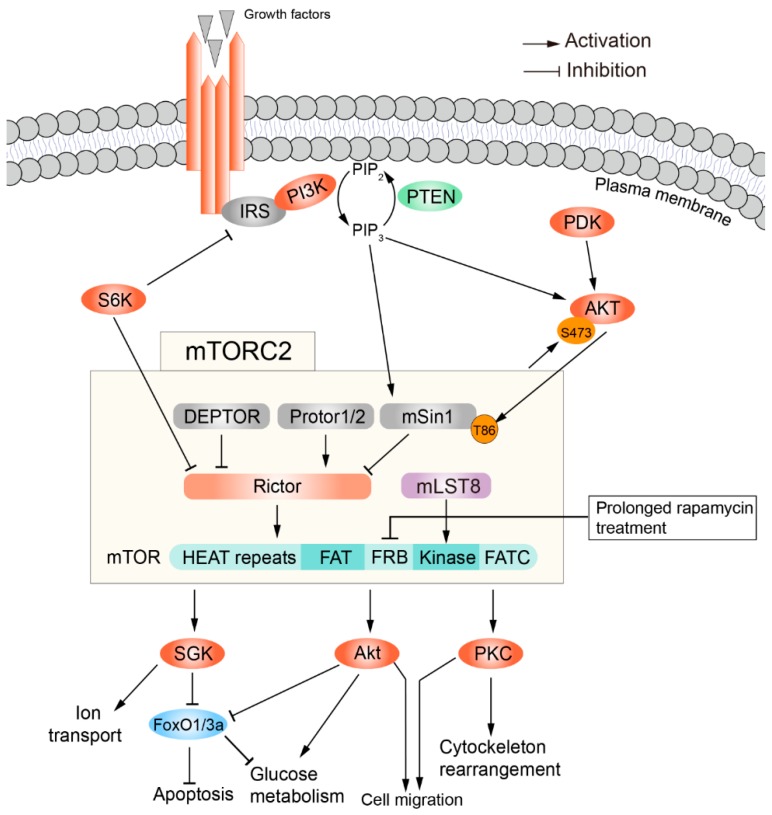
The structures, regulatory mechanism and functions of mTORC2.

The mTORC2 mainly controls cell migration through phosphorylation of the AGC (protein kinase A/G/C) protein kinase family members such as PKCα [56], PKCδ [62], PKCξ [63], PKCγ and PKCε [64], all of which regulate cell migration through modulations of various aspects of cytoskeletal remodeling. Furthermore, another important function of mTORC2 is phosphorylation and activation of Akt, which in turn phosphorylates and inhibits forkhead box O1/3a (FoxO1/3a), TSC2 and the metabolic regulator glycogen synthase kinase 3β (GSK3β) [65,66], thus promoting cell survival and proliferation. In addition, mTORC2 can phosphorylate and activate SGK1, which regulates ion transport for cell survival [67] (Figure 2). mTORC2 is also involved in cancer, Alzheimer’s disease (AD) [10,68].

## 4. Roles of mTOR in Neuronal Regeneration

The blood-brain barrier (BBB) is formed by endothelial cells, pericytes and astrocytes. These cells together form the neurovascular unit (NVU), which serves as an interface between the blood and the neural tissue. Impairment of BBB function is associated with neurodegenerative diseases [69]. The brain endothelial cells are vital for the function of BBB [70]. Brain vascular damage or occlusion can cause cerebrovascular diseases such as microbleeding, hemorrhagic stroke, and ischemic stroke. Macrophages and lymphatic vessels are important for the repair of brain blood vessels and the restoration of BBB functions [71,72]. The nervous system is comprised of the central nervous system (CNS) and peripheral nervous system (PNS) [73]. The PNS has a unique ability to regenerate [74,75,76]. However, the CNS of adult mammals including the brain and spinal cord obtains very limited regenerative capacity, which might partially result from abundant inhibitory growth factors in the CNS [77,78,79,80]. Effective therapeutic approaches are still missing for a wide variety of human neurodegenerative diseases including Parkinson's disease (PD), amyotrophic lateral sclerosis (ALS), Huntington's disease (HD), and Alzheimer's disease (AD) [81,82,83,84,85,86]. Therefore, the neuronal regeneration of the CNS of adult mammals constantly remains an important research topic, being of great significance for clinical treatment.

In general, axons after injury do not spontaneously regenerate in adult mammalian CNS because of a diminished intrinsic regenerative capacity and extrinsic growth-inhibitory factors [77,87,88]. Inhibitory factors from myelin including Nogo protein families, Oligodendrocyte myelin glycoprotein (OMgp), myelin-associated glycoprotein (Mag), ephrin B3, and transmembrane semaphorin 4D (Sema4D/CD100) block CNS axonal regrowth [79,89,90]. Chondroitin sulphate proteoglycans (CSPGs) produced by the reactive astrocytes in the glial scar become main inhibitory extracellular matrix (ECM) molecules at the lesion site of a mature CNS [90,91]. Therefore, both promotion of the intrinsic regenerative capacity and suppression of the inhibitory environment are required for efficient axon regeneration [77,92]. Either overexpression of osteopontin, IGF1 and ciliary neurotrophic factor (CNTF), or genetic inactivation of PTEN, a negative regulator of mTOR signaling, TSC1/2 and suppressor of cytokine signaling 3 (SOCS3), can promote axon regeneration. Signaling pathways like Janus kinase/signal transducers and activators of transcription (JAK/STAT3), canonical bone morphogenetic protein/drosophila mothers against decapentaplegic (BMP/Smad), non-canonical BMP pathway, Jun N-terminal kinase/p38 MAP kinase (JNK/MAPK), and mTOR signaling, have been reported to promote axonal regrowth during neuronal regeneration [16,93,94,95]. Here, we summarize the roles of mTOR in axonal regeneration of the CNS and PNS.

Injuries to the optic nerves and spinal cord have been widely used to study axonal regeneration of the CNS. mTOR signaling exhibits distinct functions in the optic nerve and spinal cord regeneration [76]. mTOR signaling is highly activated at the embryonic stage and diminished in the adult retinal ganglion cells (RGCs), suggesting the different functions at various developmental stages of RGCs. Activation of mTOR via genetic deletion of TSC1 or PTEN enhances axonal regeneration of RGCs after optic nerve injury [16]. Similarly, axon regeneration robustly occurs when PTEN is deficient in mouse cortical motor neurons [96], Drosophila sensory neurons [97], and Caenorhabditis elegans motor neurons [98]. The melanopsin/GPCR (cell-type-specific G protein-coupled receptor) signaling enhances axonal regeneration of RGCs through promotion of mTORC1 signaling. Interestingly, the regenerative activation is in a light-dependent manner [99]. Class I histone deacetylation enzyme HDACs that allow histones to wrap DNA more tightly, has been shown to repress the RGCs survival and regeneration after optic nerve injury. Dual deletions of HDAC1 and HDAC2 or HDAC3 deficiency robustly promotes RGCs regeneration [100,101]. Recently, dual functions of HDAC5 have been shown in the regeneration of dorsal root ganglions (DRGs) of the PNS [102,103]. Increases in the HDAC5 cytoplasmic localization by overexpressing the mutant HDAC5^AA^ can stimulate RGCs regenerative ability after optic nerve injury. This enhanced RGC regeneration is dependent on mTORC1 [104]. Another study revealed that Wnt10b from fibroblast-derived exosomes (FD exosomes) enhances neurite regrowth through regulation of GSK3β and TSC2 to boost mTOR activation [105]. After optic nerve injury, the mTOR is unnecessary for the initial step of RGCs to enter into the regenerative status but required for long axon regeneration under inflammatory stimulation [106]. Furthermore, the elevation of mTOR either by the overexpression of Rheb, or by double deletion of Pten and Socs3, or by Pten deletion combined with the injection of cAMP (cyclic adenosine monophosphate) analogue 4-(chlorophenylthio) adenosine (CPT)-cAMP or inflammatory molecules (oncomodulin or zymosan) can enhance RGCs growth into the brain [107,108,109,110]. The combinatory treatments after optic nerve injury are able to achieve visual functional recovery.

**Figure 3:**
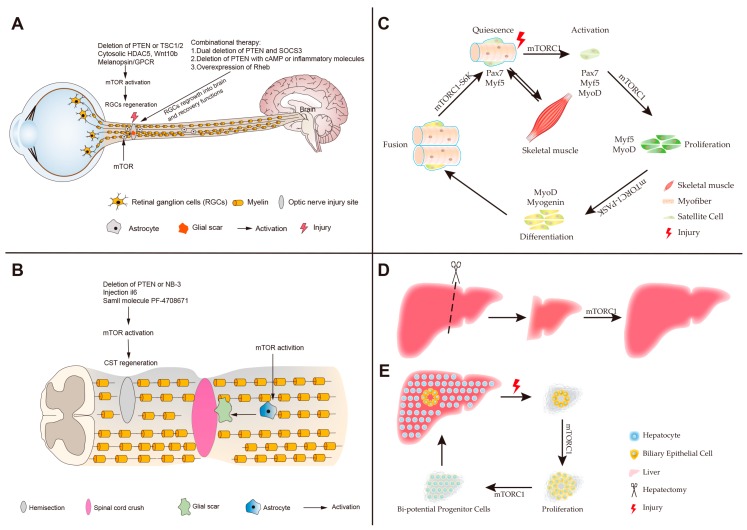
The roles of mTOR in the regeneration of neurons, muscles and the liver. (**A**) The optic nerve injury model. Activation of mTOR by deleting phosphatase and tensin homolog (PTEN) or tuberous sclerosis complex 1/2 (TSC1/2), the upstream cytosolic HDAC5 (histone deacetylase 5), Wnt10b (wingless-type MMTV integration site family, member 10b), and melanopsin/GPCR (cell-type-specific G protein-coupled receptor) robustly enhances the regeneration of retinal ganglion cells (RGCs). Combinational therapies augment the RGCs long-distance regeneration for visual function recovery through overexpression of Ras homolog enriched in brain (Rheb); the dual deletion of PTEN and suppressor of cytokine signaling 3 (SOCS3); deficiency of PTEN combined with injecting of cyclic adenosine monophosphate (cAMP) or inflammatory molecules (oncomodulin or zymosan). However, the excessive mTOR activation of astrocytes contributes to forming glial scar to inhibit axonal regeneration. (**B**) The spinal cord injury (SCI) model. In spinal cord crush model, the activated mTOR of astrocytes facilitates glial scar formation resulting in impeding the spinal cord regeneration after SCI. In the hemisection model, the stimulation of mTOR promotes the corticospinal tract (CST) regeneration post-SCI. (**C**) A schematic representation of skeletal muscle regeneration. mTORC1 stimulates satellite cells activation and proliferation, and their progenies differentiate into myoblasts under mTORC1 regulation. mTORC1 also promotes the fusion of myoblasts to form myofibers. (**D**) Partial hepatectomy (PH) model. Liver regeneration after PH is via the self-replication of existing hepatocytes, and mTORC1 regulates hepatocyte proliferation. (**E**) The severe liver injury model. Liver regeneration is via the trans-differentiation of cholangiocytes. In the process, mTORC1 regulates the proliferation of cholangiocytes and the formation of Bi-potential Progenitor Cells.

Although studies above suggest that mTOR signaling promotes RGC regeneration, Li et al. showed that mTOR is a negative regulator of RGCs survival and activates astrocytes after optic nerve injury. In the rat retinal ischemia-reperfusion (I/R) injury model, neuroprotective effects of rapamycin can reduce the loss of RGCs after optic nerve injury. Thus, the neuroprotective effects of rapamycin supply a therapeutic method for optic neurodegeneration [111]. Overall, activated mTOR robustly enhances RGCs regeneration after optic nerve injury and also promotes astrocyte activation to form a physical and biochemical barrier glial scar for inhibiting axonal regeneration (Figure 3A). Specific activation of mTOR in RGCs or inhibition in astrocytes after injury promotes RGC regrowth and regeneration, suggesting mTOR as a potential therapeutic target for optic nerve injury and neurodegenerative disease. In summary, activated mTOR enhances RGCs regeneration after optic nerve injury and also promotes astrocytes activation to form physical and biochemical barrier glial scar that could inhibit axonal regeneration (Figure 3A).

The spinal cord connects the brain with the PNS. Spinal cord injuries (SCI) are divided into traumatic and non-traumatic aetiologies [112]. Mechanisms of spinal cord regeneration are of great significance for post-SCI clinic therapy. In the animal model of SCI, the epidermal growth factor (EGF) receptor promotes transformation of inactivated astrocytes into a reactive status, which forms a glial scar to restrict neuronal recovery. The activation of astrocytes by EGF is dependent on the Rheb/mTOR pathway [113,114]. Chen et al. also showed that the PI3K/Akt/mTOR pathway contributes to the formation of glial scars by reactive astrocytes. Moreover, the overexpression of PTEN can attenuate gliosis at three days after SCI and enhances motor functional recovery [115]. In conclusion, hyperactive mTOR in astrocytes is a negative regulator for the functional recovery following SCI [114,116]. However, in different injury models like spinal cord hemisection, mTOR activation, stimulated by the deficiency of PTEN or contactin-6 (NB-3) as well as by injection with interleukin (IL-6) or small molecule PF-4708671 (an inhibitor of downstream substrate S6 kinase 1) efficiently promotes regrowth and regeneration of the corticospinal tract [117,118,119,120,121] (Figure 3B). mTOR also increases DRGs regrowth and functional recovery following PNS injury [122,123,124,125,126]. In summary, functions of mTOR in different neuronal injury models can be different or even opposing, which might be caused by mTOR activities in different cell types. Promotion of axonal regeneration can be achieved either by the inhibition of mTOR in astrocytes to attenuate glial scar formation, or by the activation of mTOR in neurons.

## 5. Roles of mTOR in Skeletal Muscle Regeneration

Muscle can quickly regenerate to recover its function after nearly complete myofiber destruction. It is important to maintain the physiological homeostasis of muscle throughout life [127]. Many studies have demonstrated that the quiescent satellite cells, which are believed to be resident muscle stem cells (MuSCs) and localized between the plasmalemma of myofibers and the basement membrane, mainly contribute to the skeletal muscle regeneration in response to muscle injury [128,129,130,131,132]. After muscle injury, satellite cells are activated to proliferate. Their progenies either maintain the satellite cells pool through self-renewal or differentiate into myoblasts expressing myogenic markers Myf5 (myogenic factor 5), MyoD (myogenic differentiation 1), myogenin and MRF4 (myogenic regulatory factor 4) [130,133] (Figure 3C). Ultimately, nascent myoblasts fuse together or fuse to existing myofibers to form new myofibers to accomplish skeletal muscle regeneration [130]. In different models of muscle injury, this process is regulated by multiple pathways such as AMPK, IGF-1/Akt, TGFβ/Smad and mTOR [127,130,133,134,135,136,137]. Here we focus on the roles of mTOR in the regulation of skeletal muscle regeneration.

In mice, conditional knockout (cKO) of *mtor* or *raptor* in MuSCs effectively inhibits activation, proliferation, and differentiation of satellite cells, which impairs skeletal muscle regeneration [133,134,135,136,137] (Figure 3C). Rapamycin treatment also confirms that mTOR inhibition indeed blocks the formation of nascent myofibers and the growth of regenerating myofibers during skeletal muscle regeneration [134,136]. Rapamycin-resistant (RR) and RR/kinase-inactive (RR/KI) experiments demonstrate that mTORC1 signaling regulates muscle regeneration through both kinase-independent and kinase-dependent mechanisms [134]. S6K1 is dispensable for the initial formation of nascent myofiber during regeneration, but its ablation impairs later muscle growth [134]. Ablation of 4EBP1 facilitates myofiber growth, but does not affect the activation of satellite cells [137]. After injury, activation of Per-Arnt-Sim domain kinase (PASK), a downstream phosphorylation target of mTORC1, phosphorylates Wdr5 to induce the expression of *myogenin* and stimulates MuSCs differentiation into myoblasts [138] (Figure 3C). The mTORC1-S6K pathway is also required for myoblasts fusion to accomplish myofiber formation during muscle regeneration [138]. micro-RNAs (miRNAs) have been reported as important modulators of myoblasts formation and fusion during muscle regeneration [139,140,141,142]. micro-RNA-1 (miR-1) stimulated by mTORC1 increases myoblast differentiation and enhances muscle regeneration through the HDAC4-follistatin axis [141]. Similar to miRNAs, long non-coding RNAs (lncRNAs) also regulate skeletal muscle regeneration. The lncRNA LINC00961 encodes a polypeptide small regulatory polypeptide of amino acid response (SPAR), which negatively regulates mTORC1 activation by interacting with lysosomal v-ATPase. The downregulation of SPAR after skeletal muscle injury activates mTORC1 to enhance muscle regeneration [143]. In contrast to mTORC1, *rictor* knockout in embryonic and adult satellite cells is ineffective in skeletal muscle regeneration [136,144]. After trauma, the presence of bone-derived and cardiac muscle-derived tissue ECM scaffolds in damaged muscle recruits more immune cells and form immune microenvironment, which facilitates muscle regeneration. Transplanted WT CD4^+^ T cells rather than *rictor*^−/−^ CD4^+^ T cells promote muscle regeneration in the *Rag1*^−/−^ mice [145]. Taken together, these studies illustrate that mTORC1, but not mTORC2, acts as a key regulator of skeletal muscle regeneration.

In muscle injury-regeneration models induced by cardiotoxin (CTX) [146], BaCl_2_ [142] and ischemia/reperfusion (I/R) [147], some pathways regulate muscle regeneration through mTOR signaling. In I/R-induced muscle injury, skeletal muscle protection by activated Sonic hedgehog (Shh) is blocked by the inhibition of AKT/mTOR/p70S6K [147]. This result implies that Shh stimulates skeletal muscle regeneration through the AKT/mTOR/p70S6K pathway. In CTX-induced muscle injury, Ca^2+^ influx flows into cells through T-type Ca^2+^ and Trpc1 channels, which enhances the activation of PI3K to activate the Akt/mTOR/p70S6K pathway and ultimately improves muscle regeneration [146]. The IGF pathway is essential for skeletal muscle regeneration [148,149]. mTORC1 inhibits miR-125b, which is a negative regulator of IGF2, thus promoting muscle regeneration after BaCl_2_ treatment [142]. Furthermore, nutmeg extract stimulates soleus muscle regeneration through IGF1-AKT-mTOR and inhibits autophagy in aged rats [150].

Muscle-mass weakness and wasting are often caused by various pathological conditions, such as sarcopenia, diabetes and chronic obstructive pulmonary disease (COPD). On average, geriatric patients with sarcopenia lose 30% of their muscle mass and 35% of myofibers [151], and have decreased muscle protein synthesis [152]. Studies on aged rodents clarify that aging decreases the muscle regenerative capacity after injury [152,153,154]. Comparing to the aged rodents, young rodents with leucine supplements strongly improve skeletal muscle regeneration. During muscle injury, both aged and young rodents activate Akt/mTOR pathway, p70S6K and 4EBP1, but the young rodents have stronger activation than aged ones. Leucine supplementation increases the activation of the PI3K/Akt/mTOR pathway, then improves muscle regeneration in aged rodents [155,156,157,158,159]. However, homeostatic maintenance of MuSCs pool is also critical to ensure muscle regeneration upon re-injury in aged muscles [160,161]. Upon repeated injuries, differentiation of MuSCs at the expense of MuSCs is promoted by mTORC1 in aged mice, which can be inhibited by rapamycin [162]. In mice, skeletal muscle atrophy can be induced by diabetes. Acupuncture with low-frequency-electric-stimulation (Acu-LFES) promotes muscle regeneration through the IGF-1-Akt-mTOR pathway [163]. The muscle regeneration stimulated by the Acu-LFES induced miR-1 may also act through the mTORC1-miR-1-HDAC4-follistatin axis [141,163]. In mice with hypoxic treatment, mTOR is inhibited by the activated AMPK or REDD1 (regulated in development and DNA response 1), both of which impair muscle regeneration [164]. Muscle injury and the decreased mTOR activity are observed in COPD patients with hypoxemia [165]. At present, there are drugs/targets to be used in clinical trials to treat muscle wasting. However, drugs targeting mTOR signaling have not been trialed yet [166], which may be of future clinical interest to inhibit muscle atrophy and promote muscle regeneration.

## 6. Roles of mTOR in Liver Regeneration

The liver possesses extraordinary regenerative capacity comparing to other internal organs [167,168,169]. Partial hepatectomy (PH) of rats and mice is one of the most widely used models to study liver regeneration [170,171]. In 1931, Higgins et al. first proposed a rat model with 2/3 hepatectomy, in which the remaining liver restores its original weight after 5–7 days of surgical resection [168] (Figure 3D). Other acute injury models include tetrachloride (CCl_4_)- and thioacetamide (TAA)-induced chemical liver injury. Remaining hepatocytes after PH or chemical liver injury accomplish liver regeneration via self-replication without the participation of progenitor cells [168,172]. However, chronic liver diseases in humans cause severe liver injury and impair hepatocyte proliferation [173]. After extreme liver damage, regeneration is achieved via cholangiocytes transdifferentiation rather than self-replication, in zebrafish [174,175] and mice [176,177] (Figure 3E). The mTOR signaling pathway mediates liver regeneration after PH and initiates the transdifferentiation of biliary epithelial cells (BECs) after extreme liver injury [178,179].

A 2/3 hepatectomy in rodents promotes the release of cytokines and growth factors [171], which simulate hepatocyte proliferation via transducing activation of PI3K/Akt signaling pathway [180]. The “pif-pocket” mutant of PDK1 showed that the PI3K-dependent PDK1 kinase specially phosphorylates Akt during regeneration after PH [181]. In mice, deletion of vitamin D3 up-regulated protein 1 (VDUP1) enhances regeneration after PH via the HGF and TGF-α responsive activation of ERK1/2 and Akt [182,183]. In VDUP1 KO mice, 70% hepatectomy significantly increases the hepatic proliferative response, in accordance with CCl_4_ treatment [183]. mTOR activated by Akt activation is well-conserved in the regulation of cell cycle and cell proliferation during liver regeneration [184] (Figure 3D). Hepatocyte proliferation in liver regeneration is regulated by cell cycle-related proteins, such as cyclin dependent kinase (CDK), cyclin D, cyclin E [185]. However, phosphorylation and activation of S6K1 by mTOR is vital to regulate the expression of cell cycle-related proteins, particularly cyclin D1, during liver regeneration [186]. After PH, rapamycin significantly reduces the rate of hepatic proliferation through inhibition of p70S6K activation, but not inhibition of p4EBP1 [187]. Meanwhile, it increases the rate of hepatic apoptosis [188] and significantly eliminates bleeding-induced hepatocyte hypertrophy [189]. After 2/3 hepatectomy, Cyclin D1 translation promoted by microRNA-21 (miR-21) enhances liver regeneration via eliminating the inhibition of Ras homolog gene family member B (Rhob) on Akt1/mTORC1 [190]. Cyclin D1 and CDK4 form an activation complex to promote liver regeneration via stimulation of G1 to S transition in hepatocytes [185]. Deletion of *sirtuin6 (sirt6)*, one of the sirtuin family of class III NAD^+^-dependent histone deacetylase, delays G1 to S phase transition and impairs activation of the Akt/mTOR pathway during liver regeneration [191]. However, SIRT1 overexpression in mice impairs liver regeneration, which can be reversed by the leucine-activated mTORC1 [192]. Genetic manipulation or chemical drugs treatment, such as inactivation of apoptosis-stimulating protein two of p53 (ASPP2) [193], *let-7* deletion [194], rosmarinic acid [195], carbamazepine [196], and panax notoginseng saponins [197], stimulate liver regeneration by activation of mTOR after PH, which is blocked by the mTOR inhibition [194,195,196]. Severe injury in PH such as a 90% hepatectomy suppresses the capacity of liver regeneration, which is accompanied by the inactive mTOR signaling [198]. In addition to hepatocyte proliferation, increases in the hepatocyte size could account for liver regeneration [181,184,199]. In liver-specific STAT3-knockout (LS3-KO) mice, although hepatocyte proliferation after injury is impaired by the decreased cyclin D1 expression, increases in hepatocyte size during early liver regeneration may occur via the activation of the PI3K/Akt/mTOR pathway [181,199].

Contributions of non-parenchymal cells (NPCs) can also be important for liver regeneration [174,176,177,179,200]. Expansion of BECs during ductular reaction (DR) is promoted by mTORC1 signaling after extreme liver injury in zebrafish and mice [179,200] (Figure 3E). Inhibition of mTORC1 remarkably suppresses the dedifferentiation of BECs and the proliferation of bi-potential progenitor cells (BP-PCs), thus leading to a reduced number of BP-PCs for re-differentiation and impairing liver regeneration after extreme injury [179,200]. Estrogen promotes liver regeneration through the activation of mTORC1 signaling in zebrafish [201]. In contrast, the *rictor* mutant only loses 20% of regenerating liver mass compared to the wild-type [179]. These studies suggest that liver regeneration is mainly mediated by mTORC1 rather than mTORC2 signaling [179].

In addition to endogenous liver regeneration, liver transplantation is also an effective way to restore liver mass and functions after liver failure or cirrhosis [202,203]. In patients, associating liver partition with portal vein ligation during staged hepatectomy (ALPPS) activates mTORC1 signaling [204]. Small-for-size mouse liver transplantation (30% grafts) significantly downregulate mTORC1 signaling and suppress liver regeneration [205,206]. However, half-size transplantation (50% grafts) increases the mTORC1 activity. 30% grafts treated with amphiregulin restore mTORC1 activation and p70S6K phosphorylation to the level of 50% grafts [206]. These studies imply that activation of mTORC1 may be a promising therapeutic approach to stimulate liver regeneration during liver transplantation.

## 7. Roles of mTOR in Intestinal Regeneration

The small intestinal epithelium is a single cell layer with rapid self-renewing and strong regenerative capabilities. The mammalian intestinal epithelium mainly includes enterocytes, Goblet cells, enteroendocrine cells, and Paneth cells [207]. In flies, esg^+^ (escargot) intestinal stem cells (ISCs) and enteroblasts (EBs) are responsible for gut homeostasis and drive the regeneration following injury [208,209]. The TSC/TOR signaling pathway regulates proliferation and maintenance of ISCs in response to nutritional conditions [162,210]. Also, mTORC1 is the main component of mTOR pathway that is decisive for the intestinal development in zebrafish [211]. Similarly, *Lgr5* marks the ISCs at the base of intestinal crypt which maintain the intestinal homeostasis and intestinal regeneration in mouse [212]. Furthermore, *Dll1*^+^ secretory progenitor cells, *Alpi*^+^ enterocyte progenitor cells, and *Lyz*^+^ Paneth cells can be induced to ISC after specific ablation of *Lgr5*^+^ ISC [213,214,215]. mTORC1 takes an pivotal part in increasing the maintenance of ISCs activity and proliferation of intestinal epithelium [216]. In addition, the cooperation of mTORC1 and SIRT1 promotes the expansion of ISCs during the calorie restriction (CR) [217]. Taken together, mTORC1 regulates the intestinal development and maintains intestinal hemostasis and ISC, in which mTOR is conserved in drosophila, zebrafish and mouse.

The intestinal epithelium, which has a powerful regenerative capacity after injury, provides a model to study ISCs, cancer, and intestinal regeneration [218]. A few studies demonstrate that mTORC1 signaling is required for intestinal regeneration and conserved in *Drosophila* and mammals. In inflammatory bowel disease (IBD) mouse models induced by 2,4,6-trinitrobenzene sulfonic acid (TNBS) or dextran sodium sulfate (DSS), mTORC1 is required for intestinal regeneration against IBD. The deficiency of Regnase-1 promotes the intestinal regeneration in the acute IBD via controlling the mTOR and purine metabolism [219]. Akt/mTOR activated by the focal adhesion kinase (FAK) is required for the Wnt/myc-mediated intestinal regeneration and tumorigenesis [218,220]. mTORC1-S6k1/2 axis, but not eiF4ebp1/2, enhances crypt regeneration after DNA damage [221]. These studies imply that activation of mTORC1 may become a therapeutic approach for IBD and other intestinal injuries [222]. Besides, the mTOR-dependent autophagy is impaired in patients with ulcerative colitis (UC), implicating mTOR also as a therapeutic target for autoimmune diseases [223].

## 8. Perspectives

In this review, we summarize the roles of mTOR in the regeneration of neurons, muscles, the liver, and the intestine, which are mainly mediated by mTORC1 rather than mTORC2 signaling. The mTORC1 signaling network may be disrupted in various diseases, such as ALS, AD, PD, COPD, sarcopenia, liver failure, and IBD, which is harmful to tissue regeneration [151,165,203,222,224,225,226]. Although regeneration and recovery of injured tissues in human may be improved by drugs promoting mTOR such as carbamazepine and nutmeg [150,196,224,225], mechanisms underlying these drugs remain largely unknown. And the curative effects of these drugs still need improvements. Additionally, the agonist and inhibitor of mTOR lack tissue specificities, like leucine and rapamycin [2,225]. For example, in addition to the inhibition of mTORC1, rapamycin can also inhibit mTORC2 during a long-term treatment [225]. A large number of studies have reported that drug delivery systems can increase tissue specificity. For example, a bone-targeted nanoparticle (NP) delivery system can carry a β-catenin agonist or GSK3β inhibitor to fractured bone with concentrated accumulation, which enhances bone regeneration [227]. A tissue-specific agonist or antagonist of mTORC1 might also be achieved with this strategy. Tissue-specific mTORC1-antagonist may effectively treat neurodegenerative diseases resulting in nerve injury, and tissue-specific mTORC1-agonist may effectively promote muscle recovery in muscle injury. Recently, sirolimus in phase 1/2 trial has been shown to effectively treat patients with systemic lupus erythematosus (SLE) against tissue injury, improving the expansion of naïve T-cell populations and decreasing CD8^+^ memory T cells. But in this process, sirolimus causes reversible oral ulcers, headaches, and cytopenia [228]. The tissue-specific inhibitor of mTORC1 may prevent these side-effects in the clinic. Except organs depicted in this review, roles of mTOR signaling in the regeneration of other organs like the pancreas, heart, and kidney are rarely reported. Ultimately, a comprehensive understanding of the mTOR signaling network during different tissue regeneration processes, and the development of new drugs for tissue-specific mTOR activation or inhibition are of great scientific and clinical interest.
